# Heavy-atom tunnelling in benzene isomers: how many tricyclic species are truly stable?†

**DOI:** 10.1039/d4sc05109b

**Published:** 2024-09-05

**Authors:** Sindy Julieth Rodríguez, Sebastian Kozuch

**Affiliations:** a Department of Chemistry, Ben-Gurion University of the Negev Beer-Sheva 841051 Israel sindyjul@bgu.ac.il kozuch@bgu.ac.il

## Abstract

The variety of possible benzene isomers may provide a fundamental basis for understanding structural and reactivity patterns in organic chemistry. However, the vast majority of these isomers remain unsynthesized, while most of the experimentally known species are only moderately stable. Consequently, there is a high probability that the theoretically proposed isomers would also be barely metastable, a factor that must be taken into account if their creation in the laboratory is sought. In this work, we studied the kinetic stability of all 73 hypothetical tricyclic benzene isomers, especially focusing on their nuclear quantum effects. With this in mind, we evaluated which species are theoretically possible to synthesize, detect, and isolate. Our computations predict that 26% of the previously deemed stable molecules are completely unsynthesizable due to their intrinsic quantum tunnelling instability pushing for their unimolecular decomposition even close to the absolute zero. Five more systems would be detectable, but they will slowly and inevitably degrade, while seven more supposedly stable systems will break apart in barrierless mechanisms.

## Abstract 2

We studied the kinetic stability of all 73 hypothetical tricyclic benzene isomers focusing on their nuclear quantum effects. Which ones may pass the quantum tunnelling instability test, and which ones will be unsynthesizable and unisolable?

## Graphical abstract

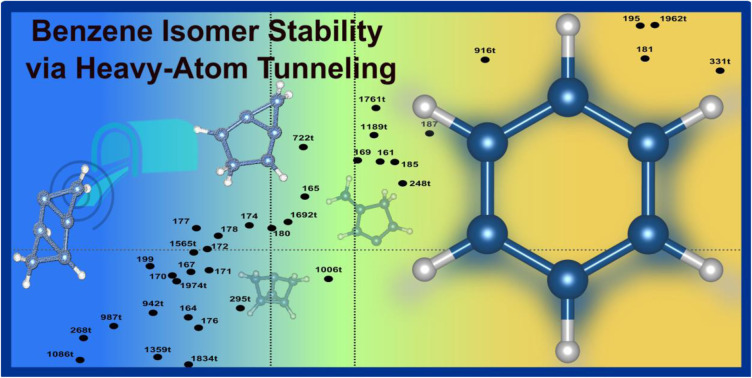

## 1 Introduction

Benzene, with the chemical formula C_6_H_6_, is the indisputable best-known aromatic compound. Its elegant symmetrical structure provides an outstanding stability, but its geometry was not originally evident, especially considering the plethora of possible isomers. While Faraday^1^ discovered and isolated benzene in 1825, it was not until 1867 that Kekule^2^ proposed the celebrated “snake seizing its tail” cyclic hexagonal structure. But this was not the only proposed structure for benzene, since the connectivity of six carbons can hugely vary. Indeed, excluding diastereomers and enantiomers, 217 theoretically stable structural isomers of benzene were recently identified by computational means, all of them of higher energy than Kekule's benzene, the ruling ring.^3,4^ These isomers were classified into five major groups: acyclic, monocyclic, bicyclic, tricyclic, and tetracyclic.

Historically, the first observed benzene isomer was Dewar's, proposed in 1869 but synthetically achieved only in 1963.^5,6^ This is a bicyclic system that proved to be only moderately stable, with a half-life of two days at room temperature (it would be less stable if not for the forbidden pericyclic reaction that converts it to benzene). Subsequently, benzvalene was synthesized in 1967;^7,8^ this tricyclic isomer has a half-life of about ten days, is stable only in solution, and is explosive in pure form. Isomers such as bicyclopropenyl and prismane have also been synthesized,^9^ the latter being stable at room temperature but decomposing at 363 K in toluene with a half-life of eleven hours. In 1990, the 1,2,3-cyclohexatriene strained isomer was produced, with a DFT computed energy of 418 kJ mol^−1^ above benzene.^10^

In 2001,^3^ the first enumerated list of possible benzene isomers appeared, and their classification into five major groups: acyclic, monocyclic, bicyclic, tricyclic and tetracyclic. A comprehensive study of the potential energy surface (PES) of C_6_H_6_ reported that of the 217 isomers, only 198 are local minima,^11^ with benzene being the global minimum by at least 125 kJ mol^−1^ compared to the following isomers. A following study expanded these numbers to 2000 local minima structures, although this included clusters of disconnected submolecules.^4,12^ Recently, a study on the magnetic properties of 198 isomers by Janda^13^ showed that only four of them have higher magnetic susceptibility anisotropy than benzene. In this work, they also reported five new isomers and left open the question of a possible correlation between energy stability and magnetization.

The wide variety of benzene isomers provides a fundamental basis for understanding structural patterns in organic chemistry, thus opening the possibility of developing and synthesizing new compounds with practical applications.^14^ But considering the large amount of species, with the vast majority of them only theoretically known,^15^ and based on the fact that most of the synthesized ones are only moderately stable, it is evident that most of the hypothetical isomers will probably be barely metastable, a factor that must be considered if someone would embark on trying to create them in the lab.

Answering the question of the hypothetical molecular stability for such a large number of systems is technically impossible with experimental means, but it can be resolved through computational quantum chemistry. However, to be comprehensive and accurate, such study must include any possible effect that can compromise the molecular stability, especially nuclear quantum effects (the main topic of this article).

The concept of molecular stability itself is ambiguous. It was roughly categorized within two categories: “viable” and “fleeting”.^16,17^ A molecule is viable if it may maintain its structure under typical chemical laboratory conditions; in contrast, “fleeting” refers only to whether the molecule is at a local minimum in the PES and therefore it might only be stable at extremely low temperatures.

However, in many cases cryogenic temperatures are not enough to prevent the rapid decomposition of a molecule due to a quantum tunnelling (QT)^18–20^ degradation mechanism, an effect that we called “quantum tunnelling instability” (QTI).^21–27^ Such an effect, an inherent property of many strained geometries, is rarely accounted for in computational studies, but it is unavoidable *in vitro*. In this sense, low barriers for unimolecular decompositions can signal difficult conditions for synthesis, detection, and conservation; but if tunnelling is considered, they may prove to be completely unattainable.

Although QT in molecules^28^ was traditionally considered relevant only for hydrogen due to its low mass, many reactions involving “heavy”-atom tunnelling (such as carbon and nitrogen) have been observed and/or theoretically predicted in the literature.^18,29–32^ In studying the inherent stability of organic molecules, most of the reactions will consist in breaking carbon–carbon bonds, and therefore such projects must carry out heavy-atom tunnelling analysis in their final stability test. This approach will be applied here to define the viability of benzene isomers.

As a first step, we studied the kinetic stability of all 73 theoretically known tricyclic benzene isomers^11,13^ against their unimolecular decomposition. We considered the semiclassical^33^ (SC) and quantum regimes to evaluate which ones are viable and theoretically possible to synthesize, detect, and isolate (at least at low temperatures). In here we will take the arbitrary stability criteria proposed by Frenklach *et al.*,^21^ where:

• “Stable” molecules are those whose half-life (*τ*_1/2_ = ln 2/*k* for unimolecular reactions) is higher than a year.

• “Detectable” if its *τ*_1/2_ is between 1 minute and 1 year (sufficient time to detect and characterize the compound).

• “Kinetically unstable” if the *τ*_1/2_ is less than 1 minute and therefore probably impossible to characterize.

• “Thermodynamically unstable” if the reaction is barrierless—that is, technically there is no reactant, as it is not even a minimum on the PES.

All these stability terms are temperature dependent (evidently a low-temperature stable molecule will be kinetically unstable at higher temperatures). For the purpose of this article, we are mostly considering the *τ*_1/2_ under deep cryogenic conditions close to the absolute zero (that is with negligible kinetic energy, and therefore with only QT as a possible mechanism), and for comparison at the commonly used liquid nitrogen temperature as well. In addition, we state the stability in a gas phase, without the influence of other possible reactants that may decompose the isomers by alternative bimolecular reactions. In other words, we check the molecules under their ideal stability conditions.

To evaluate the kinetic stability of a molecule it is necessary to know its unimolecular decomposition mechanism. To the best of our knowledge there are no comprehensive studies reporting the degradation pathways for the tricyclic isomers of benzene (or of any other group). Therefore, we divided this study into three parts:

1. First, the computation of the most probable exothermic degradation pathways for the 73 tricyclic isomers in the semiclassical regime. This was carried out by systematically stretching all the bonds that make up the molecular framework so as not to overlook possible significant coordinates in systems with complex connectivity (a very demanding process in terms of man-hours, but aided by home-developed scripts to facilitate the process). Since there are several degradation pathways per isomer, we report only the ones corresponding to the lowest activation energies. For 6 particular isomers, we reported their endothermic reaction pathway since their products were all of higher energy compared to the reactant.

2. Second, we selected the isomers with a degradation threshold energy (Δ*E*^‡^) lower than 60 kJ mol^−1^ (including zero point energy) to study their degradation pathways and rate constants including nuclear quantum effects. In all the preliminary studies on several molecules with Δ*E*^‡^ close to this value the ground-state QT included lifetimes were enormous (for example 1962t and 195 with Δ*E*^‡^ close to 60 kJ mol^−1^ have tunnel-corrected lifetimes of 10^28^ years), and therefore over this threshold energy we considered that deep tunnelling was essentially negligible.

3. Finally, for all potentially tunnelling-unstable isomers we evaluated and analyzed the kinetic stability in classical and quantum regimes to detect unexpected effects or outcomes, including products with novel geometries, multi-step degradation (“domino tunnelling”),^34,35^ or cases with tunnelling control.^36–39^

## 2 Computational methods

All structure and electronic energy computations were performed with Gaussian16 at the M06-2X/6-311G(d) level.^40,41^ This method has proven to be adequate for organic decomposition reactions,^21^ while at the same time it is fast enough for the costly tunnelling computations. To obtain more accurate threshold and reaction energies, we carried out single point energies with CCSD(T) with a complete basis set (extrapolated from cc-pVTZ and cc-pVQZ with *β* values of 5 and 3 for the SCF and post-HF correlation). In cases of open-shell singlet structures, we employed the unrestricted versions of DFT and coupled cluster. All the expressed energies include zero-point energies obtained at the M06-2X/6-311G(d) level.

For the selected isomers (part 2 above), we computed the SC degradation rate constants with canonical variational transition state theory (CVT),^42^ while the tunnelling corrections were obtained with the small curvature tunnelling (SCT) approximation.^43^ To improve the M06-2X PES with the CCSD(T) values we applied the Intrinsic Symmetry-Projected Energy (ISPE) method.^44^ Quantization of the vibrational states was included with the quantised reactant state tunnelling (QRST) approximation.^45^ Note that the SCT constants expressed here include both SC and QT contributions. The rates were obtained with Polyrate17,^46^ with Gaussrate^47^ as a connection with Gaussian. For details, an example of the Polyrate input file is included in the ESI.†

## 3 Results and discussion

### 3.1 Test set

To study the kinetic stability of the tricyclic benzene isomers we employed the comprehensive test set containing all 73 isomers obtained from two databases:

• 41 molecules found by Dinadayalane *et al.*^11^ and subsequently studied by Janda *et al.* to test for magnetic susceptibility.^13^ This database is based on graph theory, so all structures satisfy complete valence (*i.e.* all carbons have four bonds, and all hydrogens have one). In the original study the geometric optimizations were computed with B3LYP/6-31G(d).

• The remaining 32 tricyclic isomers were studied by Tokoyama *et al.*^12^ including structures with and without complete valence.^48,49^ In this work, the reported isomers were obtained using the anharmonic downward distortion following (ADDF) method. The isomers were then reoptimized at the B3LYP/6-311G(d,p) level.

We remained loyal to the molecular numbering used by these articles, with the set of isomers of interest ranging from number 159 to 203 (corresponding to the tricyclic isomers by Dinadayalane *et al.*^11^ and Janda *et al.*^13^). For the additional isomers in the Tokoyama database, we added the letter ‘t’ to the labels. Since this database is randomly organized, the numbering is not consecutive. In the ESI† we include the optimized geometries of the 73 tricyclic isomers, plus their symmetry point groups. We did not take into account isomers 168, 173, 201 and 202 since they are not proper minima (the first two according to previous studies, and the others were found here that upon optimization one is completely distorted and the other is a second order saddle point). Noteworthily, the kinetic stability of the isomers was not reported in any of the previous studies.

### 3.2 Tunnelling stability analysis

**Fig. 1:**
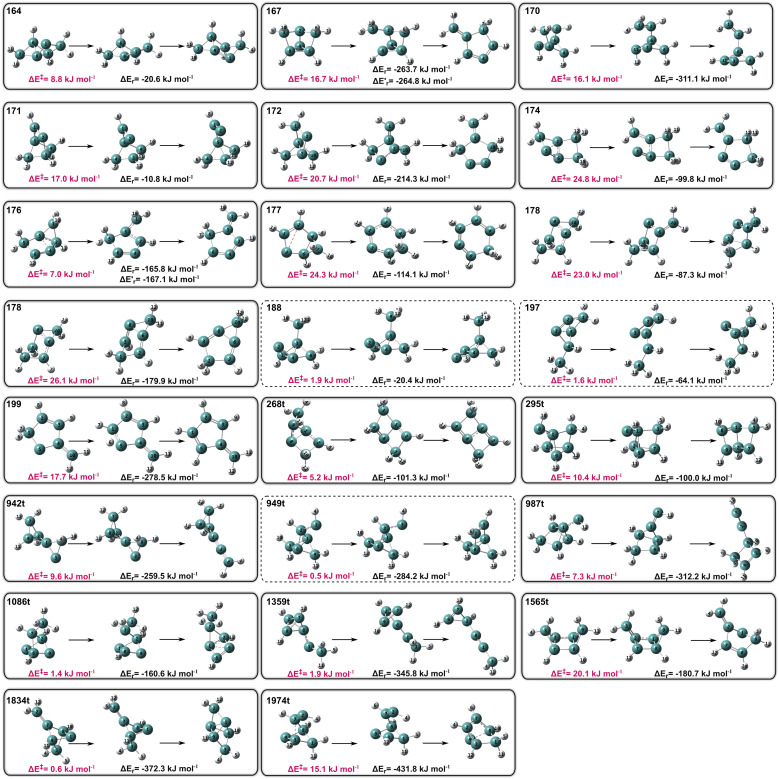
Optimized geometries of all 22 unstable tricyclic isomers, including their lowest degradation threshold (Δ*E*^‡^) and reaction energies (Δ*E*_r_) in kJ mol^−1^, including ZPE (isomer 178 has two decomposition pathways). The QT unstable isomers are highlighted in solid line boxes, and enclosed in dotted lines are the thermodynamically unstable ones.

According to our results, of the 73 tricyclic isomers, 19 of them are kinetically unstable by heavy atom tunnelling, as highlighted in solid line boxes in Fig. 1 and in Table 1. All these are technically fleeting molecules in the sense that they are minima in the PES, but in practical terms they are severely unstable by QTI. In simple terms, while all these tricyclic benzene isomers are undoubtedly synthetically complex targets, one quarter of them are technically impossible to create, detect and isolate, since they will swiftly decompose even close to the absolute zero.

**Table 1:** Tricyclic benzene isomers and their products (see Fig. 1 and S1), their energy with respect to benzene (Δ*E*_Bz_), their threshold (Δ*E*^‡^) and reaction (Δ*E*_r_) energies in kJ mol^−1^ including ZPE, imaginary frequencies at the transition state, half-lives (in s), with and without tunnelling correction (SC and QT) under deep cryogenic and liquid N_2_ conditions (corresponding to the ground state and thermally activated tunnelling), and bond length of the breaking bond in the most probable decomposition pathway. The list is ordered by half-lives under deep cryogenic conditions. Highlighted in blue are the thermodynamically unstable, in light blue the unstable, in green the detectable, and in yellow the stable systems. Products with a ★ symbol correspond to previously unreported benzene isomers

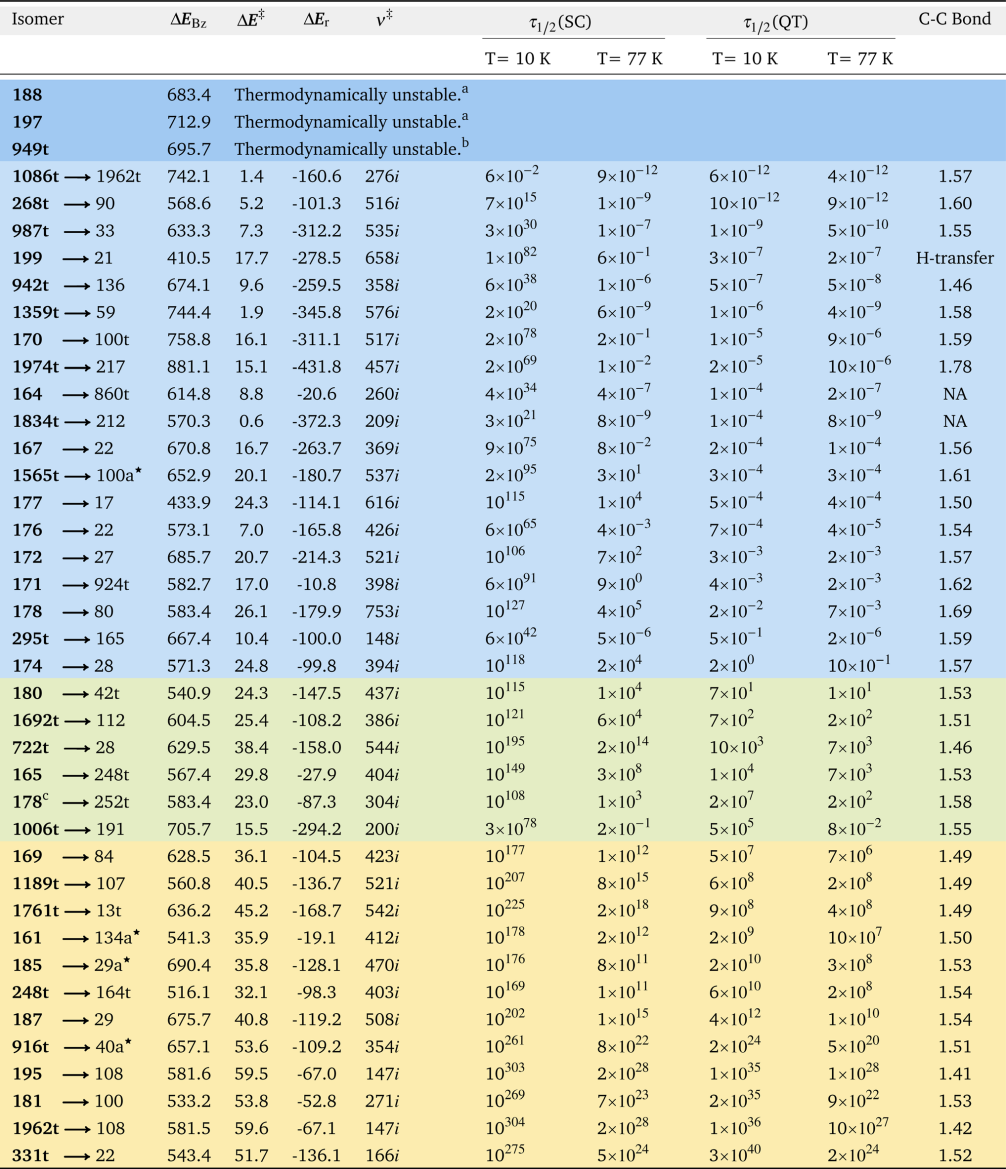

a Stable in the CCSD(T) internal electronic PES, but unstable upon addition of the ZPE correction.

b Stable according to M06-2X, but unstable according to CCSD(T).

c Second degradation path with a lower barrier.

Most of the molecular instability is caused by the breaking of C–C bonds through a tunnelling mechanism, but other outcomes are possible. For instance, one isomer (199) is a particular case of an unstable molecule due to a hydrogen-based reaction, making it the only system of the set that reacts by light atom tunnelling and not by carbon tunnelling (see below).

In studying the degradation pathways with Δ*E*^‡^ < 60 kJ mol^−1^, we found some products that correspond to new, unidentified,^11–13^ isomers of the monocyclic group, indicating that the sets may be comprehensive, but they are not complete. We therefore report here these four new structures, which we term 29a, 40a, 100a, and 134a, obtained from the decomposition of species 185, 916t, 1565t, and 161, respectively (the numbering is consistent with the database of Dinadayalane^11^ and Janda^13^ based on the number of rings, with the “a” to distinguish the new isomers).

**Fig. 2:**
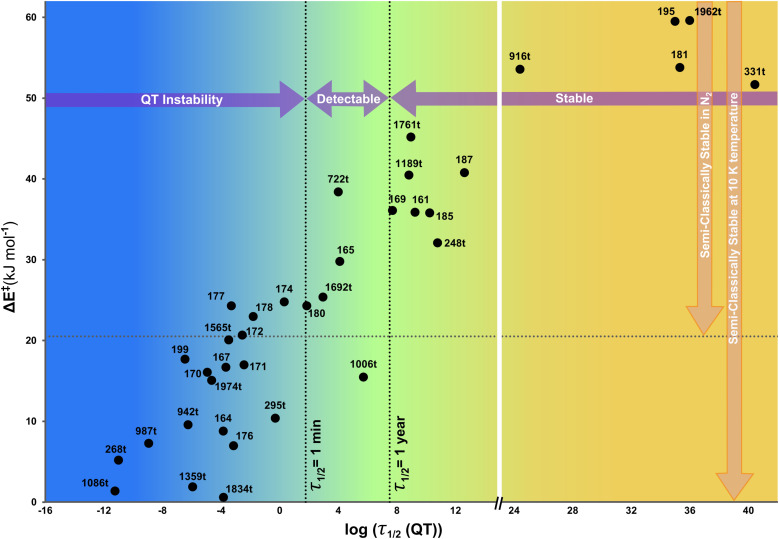
Semi-Classical/Quantum Tunnelling (SC/QT) graph^21^ for the tricyclic benzene isomers with decomposition threshold energies below 60 kJ mol^−1^ (see Table 1). The vertical axis depicts the threshold energies, directly connected with the species' SC stabilities (the horizontal dotted lines mark the threshold energies at which the molecules would be SC stable at boiling N_2_ temperature or at 10 K). The horizontal axis indicates the “real” stability including QT effects under deep cryogenic conditions (for guidance the vertical dotted lines mark characteristic half-lives). In essence, systems in the upper-left quadrant would be wrongly considered stable if QT is neglected.

**Fig. 3:**
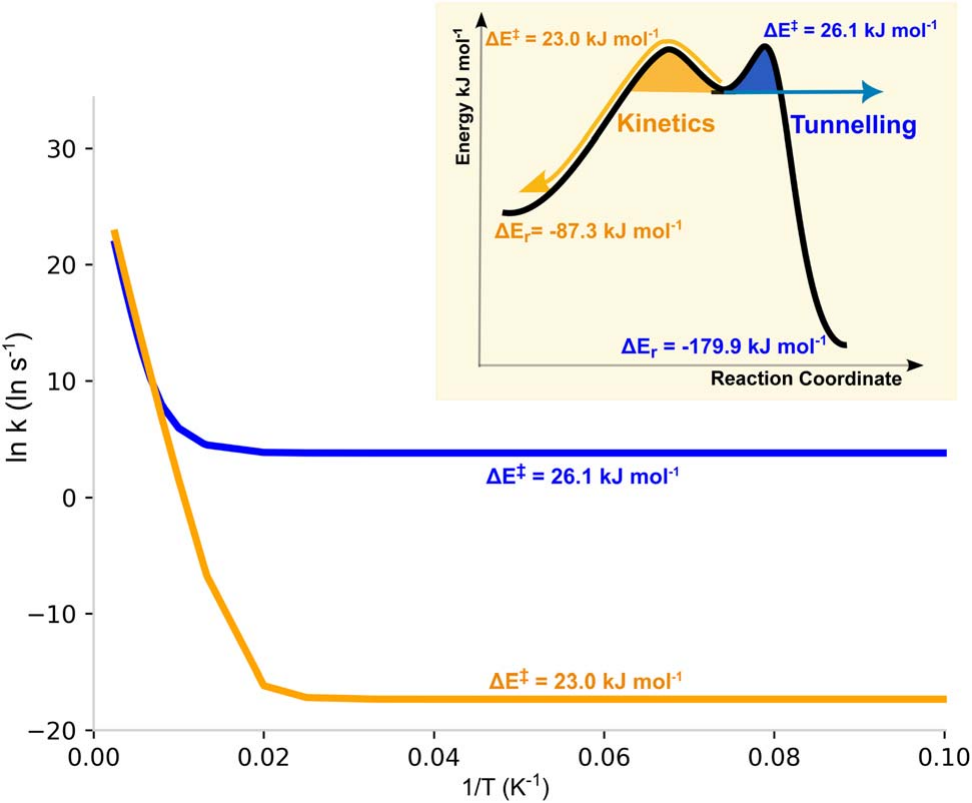
Arrhenius plot for the two pathways of the decomposition of isomer 178. The inset shows a schematic of the thermodynamic/kinetic/tunnelling control of this species.^36,38^

**Fig. 4:**
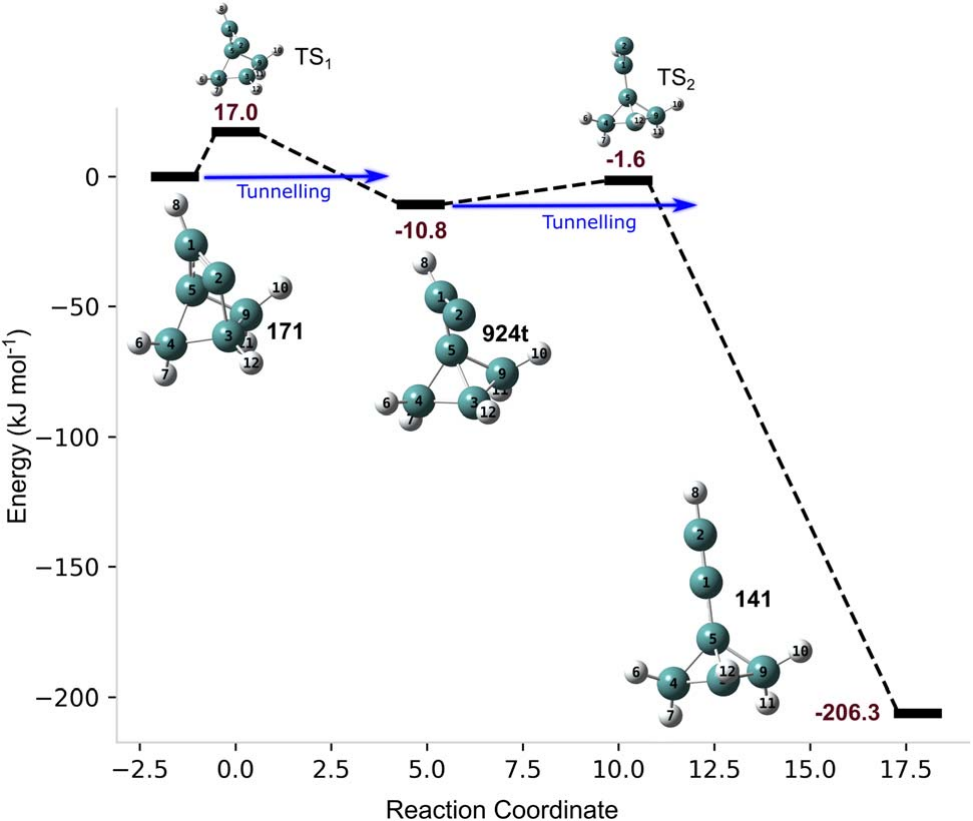
Potential energy surface for the stepwise, “domino tunnelling”^34,50^ degradation pathway from isomer 171, and their relative energies in kJ mol^−1^.

For all the tricyclic species (see Fig. S1 in the ESI†), we chose the degradation pathways with the lowest threshold energies, of which only six correspond to endothermic reactions (namely 160, 166, 175, 200, 420t, and 581t). For these we did not consider the QTI mechanism, since there is no state on the product side to carry out ground state deep tunnelling. The mostly exothermic decompositions indicate that this subset is particularly energetic, consistent with the strong strains of the three rings' structures. Most of the degradations happen at the close-shell singlet PES, an unusual outcome considering that they are predominantly C–C bond breaking (1586t and 196 are the exceptions, with open-shell singlet transitions states). 39 of the 67 isomers with exothermic decompositions have Δ*E*^‡^ < 60 kJ mol^−1^, and therefore these were the studied cases for QTI from their ground state (close to the absolute zero) and enhanced by thermally activated tunnelling (at higher temperatures), as shown in Table 1. Fig. 2, the “SC/QT graph”,^21^ visually depicts for all these systems their expected stability: in the *Y*-axis the threshold energy (a measure of SC stability) *vs.* the stability including QT in the *X*-axis (the logarithm of the half-life at 10 K computed with SCT). Of these 39 systems we can observe that:

• Beyond the four cases previously found to be thermodynamically unstable by DFT, three more hypothetically stable isomers in the M06-2X internal energy surface actually are thermodynamically unstable according to CCSD(T) including ZPE, decomposing in barrierless reactions. These isomers are marked with dashed lines in Fig. 1.

• As stated above, 19 of the supposedly stable or fleeting systems are kinetically unstable. They are not detectable according to our criteria, with *τ*_1/2_(QT) < 1 minute even close to 0 K. While theoretically possible to synthesize them, in practice it will be extremely difficult to do so, and essentially impossible to prove their fleeting existence. And unless extremely challenging laboratory setups are developed (such as with colossal static electric fields),^24^ the isolation of such species will be impossible.

• Five systems can be categorized as detectable (1 minute < *τ*_1/2_(QT) < 1 year). They may be synthesizable and in principle possible to be observed, but it will be futile to try to keep them for a significant time, as the ground state tunnelling will sooner or later lead to their decomposition.

• Twelve tricyclic molecules we deem stable, with the possibility to isolate them for long periods of time at low enough temperatures (how long and at which temperature is system dependent). These can be added to the list of 34 molecules with higher than 60 kJ mol^−1^ threshold energies, which were already considered stable.

• As cited above, our tunnelling computations predict that isomer 199, with an extremely short half-life of 3 × 10^−7^ s, is a case of light atom tunnelling, where a hydrogen is transferred from one sp^3^ carbon atom to a carbene (see Fig. S1 ESI†). The higher barrier and longer trajectory compared to other system's degradations are compensated by the smaller mass of the tunnelling determining atom.^21^ This can occur in carbene species, where the incomplete octet is fulfilled after the H-transfer creating a π bond. The reaction produces a monocyclic isomer of *C*_2v_ symmetry, an already documented stable isomer (see the ESI†). This indicates that carbon–carbon bond splitting indeed is the main decomposition mechanism for these species, especially when having stretched or strained bonds, but alternative pathways are also possible when the instability is brought by other effects.

• Isomer 178 was particularly interesting in terms of its tunnelling control^36,38^ of the degradation. According to our results, this molecule has two possible degradation pathways, with activation energies of 23 and 26 kJ mol^−1^ (see Fig. 1 and Table 1). Semi-classically the reaction will preferentially follow the former, with a temperature-dependent selectivity defined by exp(3 kJ mol^−1^/*RT*). However, the QTI also depends on the reduced masses and especially on the barrier width,^39^ causing that for this system the higher-barrier mechanism will be preferred. Therefore, at high *T* the reaction will work by classical kinetic control, but at low *T* the tunnelling control will prevail; the crossover will occur at circa 150 K (see Fig. 3 and the ESI†). As can be seen in Table 1, at 10 K the QTI goes through the higher barrier with a preference of five orders of magnitude (*τ*_1/2_(QT) = 0.02 s *vs.* 70 years). Noteworthily, the higher barrier pathway will also be technically preferable in the long-run by thermodynamic control.

• 171, as one of the unstable systems, swiftly decomposes to the bicycle compound 924t in a matter of milliseconds. Upon inspection of the latter, we found that it further decomposes with Δ*E*^‡^ = 9.3 kJ mol^−1^ with *τ*_1/2_(QT) = 10 s at 10 K (not shown in Table 1). This type of sequential quantum tunnelling phenomenon was called “domino tunnelling”.^34,50^ Note that while both reactions occur by tunnelling, the former is three thousand times faster despite having a 7.7 kJ mol^−1^ higher barrier, again showing the inescapable influence of the barrier width. Fig. 4 shows the PES for this degradation process.

• In Table 1 we show the breaking C–C bond length of each molecule. Not surprisingly, of the 19 unstable compounds (blue region of Fig. 2), 15 exhibit degradation occurring by cleavage of the longest bond, which tends to be significantly longer than the average C–C single bond (1.54 Å). Also not surprisingly, the average distance between the broken C–C bonds in the unstable isomers is significantly longer than that in the stable ones (1.58 Å for unstable, 1.53 Å for detectable, and 1.50 Å for the stable compounds). While this suggests that stretched C–C bond lengths can be used as evidence for future QTI, this is not the only factor in play. This can be observed from the lack of correlation when plotting the bond length with respect to the threshold energy or the logarithm of the half-lives (*R*^2^ ≈ 0.3). For instance, the unstable isomer 942t involves the cleavage of a very short C–C single bond (1.46 Å), probably caused by the characteristic short bonds and large ring strain of spiropentane-like structures. Moreover, stable compounds with extremely long C–C bonds of up to 1.8 Å have been observed,^51–54^ indicating that the sole use of this parameter to predict QTI can bring both false positives and false negatives. The four compounds that do not ascribe to the longest-bond breaking rule are the above-cited 942t, 199 which reacts by H-transfer, and 164 and 1834t carbenes, which suffer from geometrical rearrangements without breaking bonds.

## 4 Conclusions

In this work, we carried out a computational analysis of the kinetic stability of the 73 theoretically studied tricyclic isomers of benzene. In particular, we examined the influence of carbon nuclei quantum effects to evaluate which compounds are theoretically possible to be synthesized, detected, and isolated (at least at deep cryogenic temperatures), or if they would suffer from quantum tunnelling instability. Our results showed that of the total number of tricyclic isomers,

• Seven actually are not local minima and will decompose in a barrierless mechanism. Four are not stable on the DFT internal energy surface, and three more when including ZPE and correcting energies with CCSD(T).

• 26% of the hypothetical molecules would be kinetically unstable and probably undetectable due to the tunnelling effect, according to our half-life time threshold criteria (*τ*_1/2_(QT) < 1 minute). If we disregard this effect, these compounds would have been considered stable at temperatures roughly defined by the semiclassical transition state theory.

• Five more compounds would be experimentally detectable (1 minute < *τ*_1/2_(QT) < 1 year), but it will be futile to try to preserve them at low *T* for a significant amount of time. Quantum tunnelling from the ground state will inevitably decompose them.

Some important observations and generalizations found were that:

• Most of the QT decomposition reactions occur due to molecular instability caused by cleavage of the longest C–C bonds. However, we found one isomer that reacts due to hydrogen-transfer (the only case of light atom tunnelling), two reactions were just molecular rearrangements without any bond-breaking, and one where the most labile C–C bond actually was a very short but ring-strained bond.

• One of the isomers depicts a tunnelling-control behaviour, with two distinct degradation pathways of different threshold energies. At high temperatures, the reaction follows a classical kinetic control going through the lowest barrier. But at low *T* tunnelling predominates, and the reaction occurs through the higher but narrower barrier. This predicts a totally different selectivity depending on the temperature.

• Another isomer shows a sequential degradation pathway, where the first QTI product is also unstable, degrading into a subsequent QTI product. This quantum phenomenon is known as “domino tunnelling”.

These results on the kinetic stability of benzene isomers can serve as an example of the importance of considering quantum tunnelling in the stability assessment of hypothetical, still unknown molecules. We emphasize here the value of computational quantum chemistry and especially the attention on nuclear quantum effects, in any attempt to synthesize new, potentially strained molecules.

## Author contributions

SK: idea, writing and data analysis. SJR: all the computations, writing and data analysis.

## Conflicts of interest

There are no conflicts to declare.

## Supplementary Material

SC-015-D4SC05109B-s001

## Data Availability

Figures of all the reactions have been uploaded as part of the ESI†. All the XYZ geometries and Polyrate input file example are available on the ioChem-BD platform for computational chemistry and materials science teams, at the following link: https://iochem-bd.bsc.es/browse/handle/100/323394.
