# Supplementary material for: Heavy-atom tunnelling in benzene isomers: how many tricyclic species are truly stable?
Source: Chem Sci. 2024 Sep 5;15(41):17064–72. doi: 10.1039/d4sc05109b (PMC11428002; doi:10.1039/d4sc05109b)
Supplement: SC-015-D4SC05109B-s001 [file SC-015-D4SC05109B-s001.pdf]

Supplementary Information (SI) for Chemical Science.
This journal is © The Royal Society of Chemistry 2024

Heavy-Atom Tunnelling in Benzene Isomers: How Many Tricyclic Species are Truly Stable?

Supplementary Information

S.J. Rodríguez *[a], **S. Kozuch** *[a]

[a] Department of Chemistry, Ben-Gurion University of the Negev, Beer-Sheva 841051, Israel
* Corresponding authors: sindyjul@post.bgu.ac.il; kozuch@bgu.ac.il

Electronic Supplementary Information (ESI) available on the ioChem-BD platform for computational chemistry and materials science teams, at the following link: https://iochem-bd.bsc.es/browse/handle/100/323394

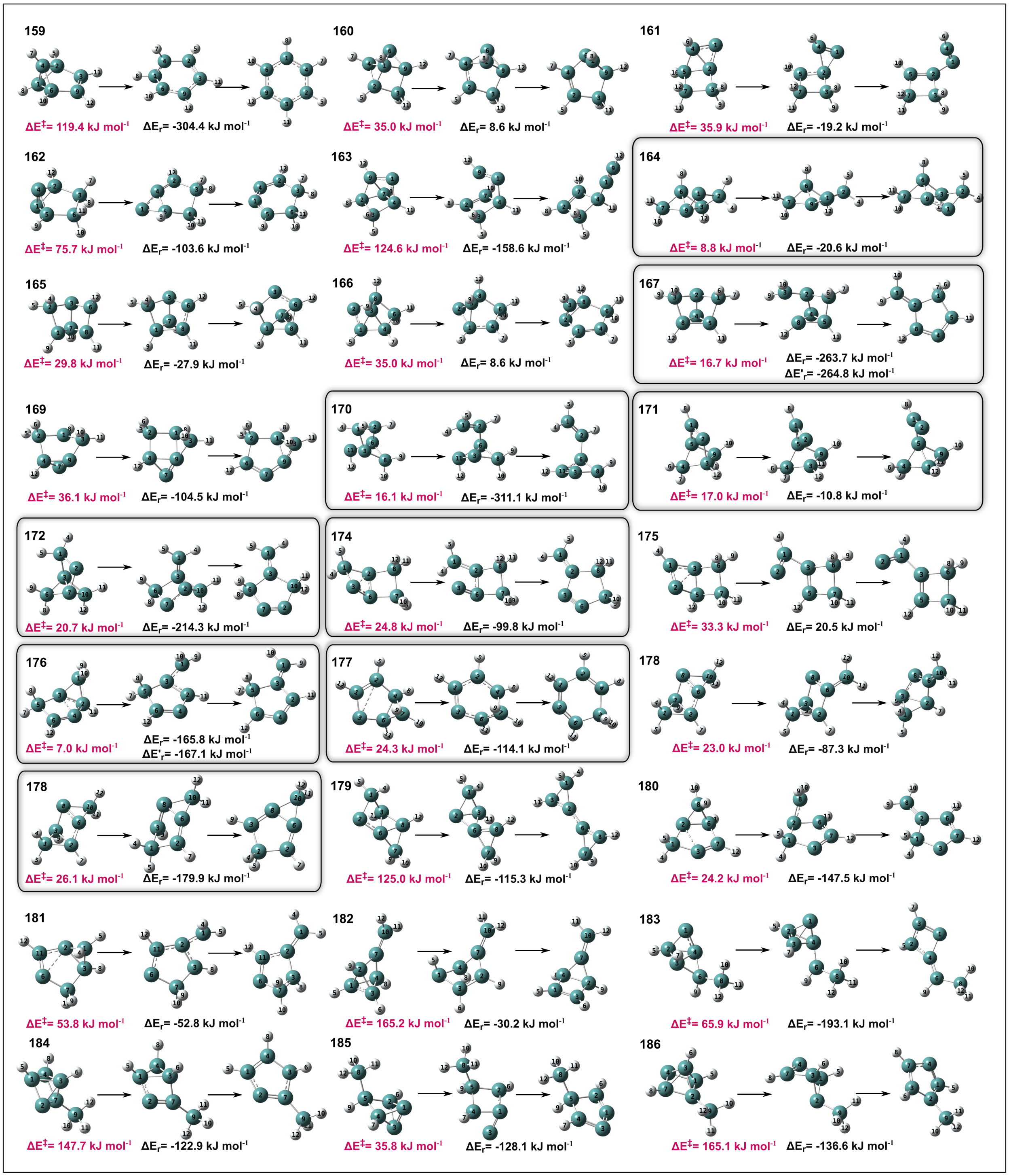

Fig. S1. Optimized geometries of all the 73 tricyclic isomers, including their lowest degradation threshold ($\Delta E^{\ddagger}$) and reaction energies (ΔE_r) in kJ mol^{-1}, including ZPE (isomer 178 has two decomposition pathways). The QT unstable isomers are highlighted in solid line boxes, and enclosed in dotted lines are the thermodynamically unstable ones. "OSS" indicates open-shell singlet species.

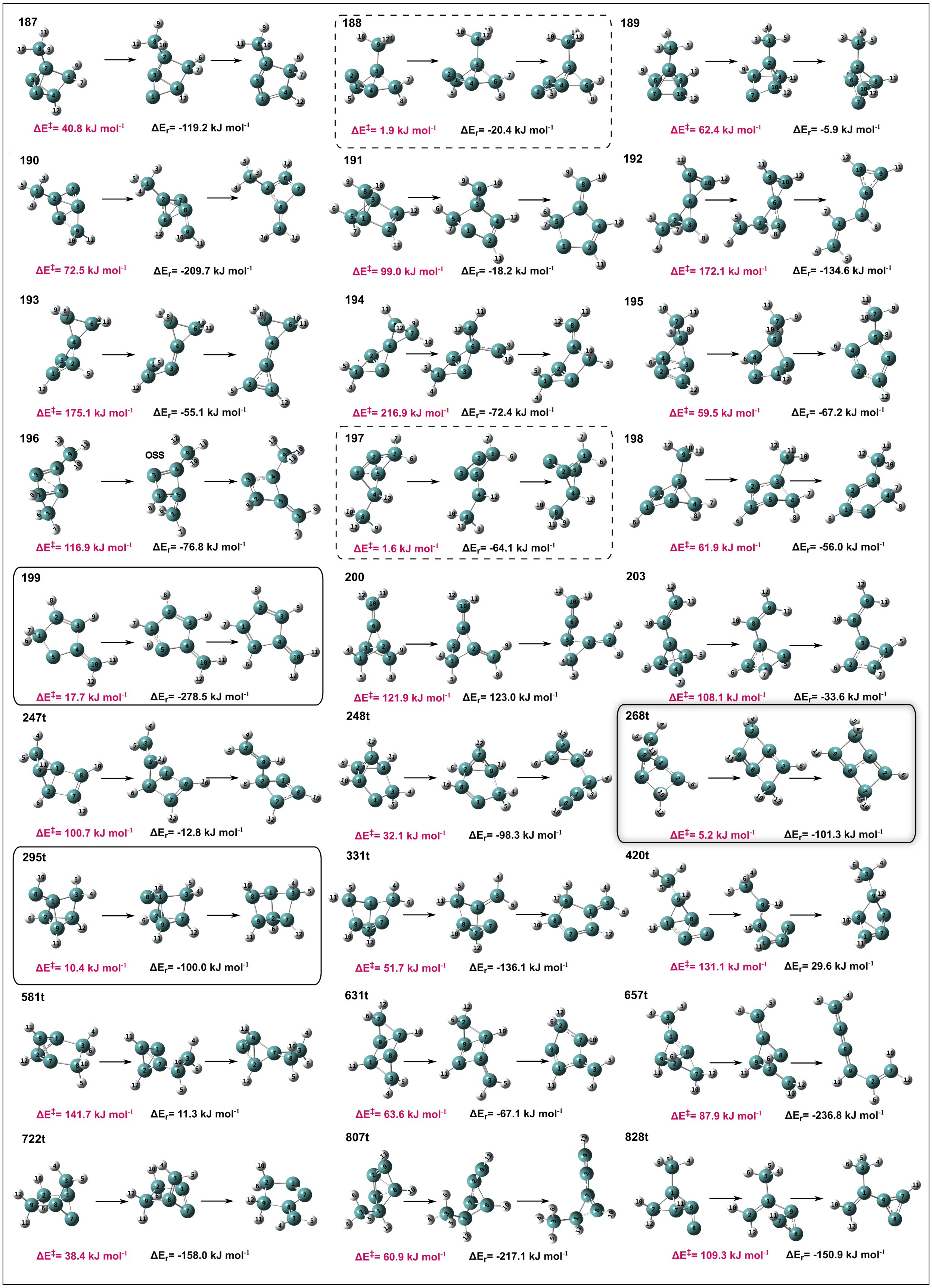

Fig. S1. Continued

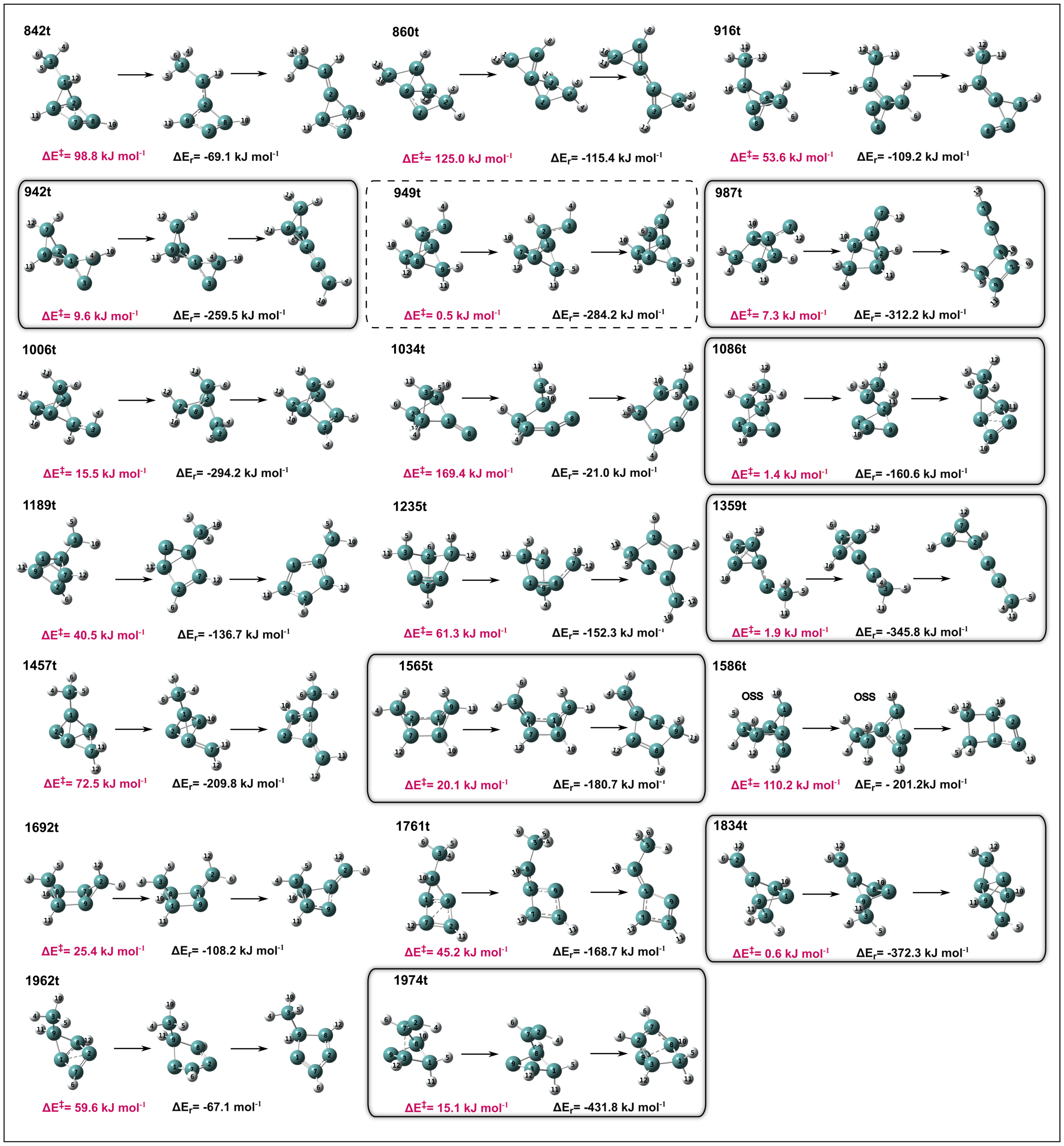

Fig. S1. Continued
